# Hinokitiol Selectively Enhances the Antibacterial Activity of Tetracyclines against Staphylococcus aureus

**DOI:** 10.1128/spectrum.03205-22

**Published:** 2023-03-21

**Authors:** Chun-Yan Le, Yu-Jian Ye, Jian Xu, Lei Li, Xi-Qing Feng, Ni-Pi Chen, Bing-Qi Zhu, Zhi-Shan Ding, Chao-Dong Qian

**Affiliations:** a College of Life Sciences, Zhejiang Chinese Medical University, Hangzhou, China; b Department of Dermatology, Third People’s Hospital of Hangzhou, Hangzhou, China; c College of Medical Technology, Zhejiang Chinese Medical University, Hangzhou, China; d Institute of Molecular Medicine, College of Life Science, Zhejiang Chinese Medical University, Hangzhou, China; Keck School of Medicine of the University of Southern California

**Keywords:** antimicrobial activity, hinokitiol, *Staphylococcus aureus*, tetracycline, synergy

## Abstract

The increasing prevalence of antibiotic resistance causes an urgent need for alternative agents to combat drug-resistant bacterial pathogens. Plant-derived compounds are promising candidates for the treatment of infections caused by antibiotic-resistant bacteria. Hinokitiol (β-thujaplicin), a natural tropolone derivative found in the heartwood of cupressaceous plants, has been widely used in oral and skin care products as an antimicrobial agent. The aim of this work was to study the synergy potential of hinokitiol with antibiotics against Staphylococcus aureus, which is an extremely successful opportunistic pathogen capable of causing nosocomial and community-acquired infections worldwide. The MIC was determined by the broth microdilution method, and the effect of combinations was evaluated through fractional inhibitory concentration indices (FICI). The mechanism behind this synergy was also investigated by using fluorescence spectroscopy and high-performance liquid chromatography (HPLC). The MICs of hinokitiol alone against most S. aureus strains were 32 μg/mL. Selectively synergistic activities (FICIs of ≤0.5) were observed for combinations of this phytochemical with tetracyclines against all tested strains of S. aureus. Importantly, hinokitiol at 1 μg/mL completely or partially reversed tetracycline resistance in staphylococcal isolates. The increased accumulation of tetracycline inside S. aureus in the presence of hinokitiol was observed. In addition, hinokitiol promoted the uptake of ethidium bromide (EB) in bacterial cells without membrane depolarization, suggesting that it may be an efflux pump inhibitor.

**IMPORTANCE** The disease caused by S. aureus is a public health issue due to the continuing emergence of drug-resistant strains, particularly methicillin-resistant S. aureus (MRSA). Tetracyclines, one of the old classes of antimicrobials, have been used for the treatment of infections caused by S. aureus. However, the increased resistance to tetracyclines together with their toxicity have limited their use in the clinic. Here, we demonstrated that the combination of hinokitiol and tetracyclines displayed synergistic antibacterial activity against S. aureus, including tetracycline-resistant strains and MRSA, offering a potential alternative approach for the treatment of infections caused by this bacterium.

## INTRODUCTION

The extended use and misuse of antibiotics in humans and animals has led to widespread emergence of antibiotic-resistant bacteria. Infections caused by drug-resistant pathogens are usually difficult to treat and account for a substantial number of deaths each year. It was reported that there were an estimated 4.95 million deaths associated with antimicrobial resistance in 2019, including 1.27 million deaths attributable to antibiotic-resistant bacteria (1). With the rapid spread of drug resistance among bacteria and the lack of new antibiotics being brought onto the market, alternatives need to be found to deal with drug-resistant infections. Over the past decades, much attention has been focused on plant-derived compounds due to their structural diversity and direct or indirect antibacterial activities against drug-resistant bacteria (2, 3). Certain plant compounds have been found to exert antibacterial activity via different mechanisms of action compared to traditional antibiotics and therefore can enhance the efficacy of existing antibiotics. For example, resveratrol has been used as an ATP synthase inhibitor to enhance the bactericidal activity of aminoglycoside antibiotics (4), while totarol exhibited a potent capacity to reverse resistance through inhibiting multidrug efflux pump activity in Staphylococcus aureus (5).

Hinokitiol (β-thujaplicin, 2-hydroxy-4-isopropyl-2,4,6-cycloheptatrien-1-one), one of the natural tropolone compounds found in the wood of cupressaceous plants, is a bioactive substance well known for its broad antimicrobial activity. It shows significant efficacy against Schistosoma mansoni, Chlamydia trachomatis, fungi, and Gram-negative and Gram-positive bacteria (6–10). It is noted that some bacteria are very sensitive to this active compound, regardless of antimicrobial resistance. The MICs of hinokitiol against Streptococcus mutans, Porphyromonas gingivalis, antibiotic-resistant Streptococcus pneumoniae isolates, and antibiotic-susceptible S. pneumoniae have been determined to be 0.3, 1.0, 0.3 to 1.0, and 0.5 μg/mL, respectively (10). However, S. aureus is less sensitive to hinokitiol than other bacteria, with MIC values ranging from 30 to 50 μg/mL (10). In a mouse pneumonia model, repeated intratracheal but not intraperitoneal administration of hinokitiol suppressed bacterial pneumonia induced by macrolide-resistant pneumococci (11). Moreover, topical application of hinokitiol resulted in a reduction in the number of S. aureus cells on the human skin surface and an improvement in skin condition after treatment (12). On the other hand, hinokitiol (10 to 300 μg/mL) did not exhibit any significant cytotoxicity toward the human gingival epithelial cell line Ca9-22, pharyngeal epithelial cell line Detroit 562, human umbilical vein endothelial cells, or human gingival fibroblasts (10). The acute intraperitoneal LD_50_ (50% lethal dose) of hinokitiol was determined to be 191 mg/kg for male ddY mice, while the oral LD_50_ of this phytochemical was reported to be 760 mg/kg for mongrel mice (13). More importantly, no treatment-related adverse effects were noted in survival rate, body weights, food consumption, or clinical signs, when F344 rats were fed a diet containing 0.005 to 0.05% hinokitiol for 52 or 104 weeks (14). Due to its broad-spectrum antimicrobial activity and low toxicity (10, 13, 14), hinokitiol has been widely used in consumer oral care products and as an antimicrobial hand-washing solution (15).

The mode of action of hinokitiol is suggested to be related to its potent metal-chelating capacity, which induces the production of reactive oxygen species and inhibits respiratory chain function dependent on iron-containing enzymes (16, 17). Although hinokitiol’s antibacterial activity has been extensively studied, there are few reports on the efficacy of the hinokitiol plus antibiotics combination. Interestingly, a recent study indicated that treating tetracycline-resistant Escherichia coli with hinokitiol selected for loss of the resistance gene encoding the TetA efflux pump (18). This finding prompted us to investigate the antibacterial effect of the combination of hinokitiol with traditional antibiotics. In the present study, we demonstrated that hinokitiol selectively enhanced the antibacterial activity of tetracycline antibiotics against S. aureus *in vitro*. In addition, we found that hinokitiol increased the intracellular accumulation of tetracycline and inhibited the efflux of ethidium bromide (EB) in S. aureus cells.

## RESULTS

### Hinokitiol displays antibacterial activity against tetracycline-sensitive and -resistant S. aureus.

In order to assess the combination effect of hinokitiol and tetracycline against different bacterial species, the MIC values of hinokitiol or tetracycline alone were determined. As shown in Table 1, hinokitiol alone displayed moderate antimicrobial activity against E. coli, Bacillus subtilis, and all tested S. aureus strains with MIC values in the range of 8 to 32 μg/mL. However, paradoxical inhibition of some S. aureus strains (ATCC 29213, ZY-4, and ZY-5) occurred at concentrations below the MIC for hinokitiol. Similar results were reported in the study by Arima et al. (12). In order to understand this phenomenon, the MICs of hinokitiol against three strains of S. aureus were determined under different pHs and inoculum sizes. As shown in Table 2, the MICs of hinokitiol were not changed under different conditions of medium pH and inoculum size. Considering that the antimicrobial activity of hinokitiol is related to its metal-chelating ability, we then tested the effects of Fe^3+^, Ca^2+^, and EDTA on the antibacterial activity of this phytochemical (Table 3). The addition of Ca^2+^ or Fe^3+^ to Mueller-Hinton (MH) broth has no effect on the MICs of hinokitiol against S. aureus. However, in the presence of Fe^3+^, the growth of ATCC 43300, ZY-1, or ZY-5 was completely inhibited by 2 or 4 μg/mL hinokitiol. Interestingly, the addition of 0.2 mM EDTA inhibited the paradoxical growth phenomenon of all tested S. aureus strains to hinokitiol, suggesting that the paradoxical phenomenon of hinokitiol against S. aureus may be related to the concentration of metal ions, especially Fe^3+^, in the culture medium.

**TABLE 1:** MICs of hinokitiol and tetracycline against standard strains and clinical isolates^*a*^

Bacterial strain	MIC (μg/mL)	
	Hinokitiol	Tetracycline
S. aureus ATCC 43300	32	0.25
S. aureus ATCC 29213	32 (4, 2, no growth)	0.25
S. aureus ATCC 25923	8	0.25
S. aureus ZY-1 (MRSA)	32	16
S. aureus ZY-2	32	32
S. aureus ZY-3	32	32
S. aureus ZY-4	32 (4, 2, no growth)	32
S. aureus ZY-5	32 (2, no growth)	0.25
S. aureus ZY-6	32	32
B. subtilis 168	32	4
E. coli ATCC 35218	16	0.5

a 4, 2, no growth means the growth of bacteria was inhibited by 2 or 4 μg/mL of hinokitiol.

**TABLE 2:** MICs of changing pH and inoculum size on hinokitiol activity against S. aureus^*a*^

Influence factor	MIC (μg/mL)		
	Staphylococcus aureus		
	ATCC 43300	ZY-1	ZY-5
pH of medium			
6.0	32	32	32 (2, no growth)
6.5	32	32	32 (2, no growth)
7.0	32	32	32 (2, no growth)
7.5	32	32	32 (2, no growth)
Inoculum size (CFU/mL)			
10^4^	32	32	32 (2, no growth)
10^5^	32	32	32 (2, no growth)
10^6^	32	32	32 (2, no growth)

a 2, no growth means the growth of bacteria was inhibited by 2 μg/mL of hinokitiol.

**TABLE 3:** MICs of the effect of Fe^3+^, Ca^2+^, and EDTA on the antibacterial activity of hinokitiol

Influence factor	MICs (μg/mL)		
	Staphylococcus aureus		
	ATCC 43300	ZY-1	ZY-5
Ca^2+^ (μM)			
0	32	32	32 (2, no growth)
625	32	32	32 (2, no growth)
1,250	32	32	32 (2, no growth)
Fe^3+^ (μM)			
50	32 (2, 4, no growth)	32 (2, 4, no growth)	32 (2, 4, no growth)
100	32 (2, 4, no growth)	32 (2, 4, no growth)	32 (2, 4, no growth)
EDTA (mM)			
0.2	32	32	32
Fe^3+^ (μM)^*a*^			
50	32	32	32
100	64	32	32

a MH medium contains 0.2 mM EDTA. 2, 4, no growth means the growth of bacteria was inhibited by 2 or 4 μg/mL of hinokitiol.

It is worth mentioning that five clinical isolates of S. aureus sensitive to hinokitiol were resistant to tetracycline (≥16 μg/mL). Among them, ZY-1 is a methicillin-resistant S. aureus (MRSA) resistant to most of the antibiotics tested, including oxacillin, gentamicin, levofloxacin, ciprofloxacin, erythromycin, clindamycin, rifampicin, and tetracycline. The susceptibilities of clinical isolates of S. aureus to different antibiotics were determined and are summarized in Table 4. Although hinokitiol showed antibacterial activity against tetracycline-sensitive and -resistant S. aureus, its MICs were higher than those of commercial antibiotics on sensitive pathogens (generally less than 1 μg/mL), implying that it should be used in conjunction with other drugs to combat S. aureus infection.

**TABLE 4:** Susceptibility of clinical isolates to different antibiotics

Antibiotic	Susceptibility^*a*^ of isolate to Staphylococcus aureus (μg/mL)					
	ZY-1	ZY-2	ZY-3	ZY-4	ZY-5	ZY-6
Tetracycline	R (≥16)	R (≥16)	R (≥16)	R (≥16)	S (≤1)	R (≥16)
Tigecycline	S (0.25)	S (≤0.12)	S (≤0.12)	S (≤0.12)	S (≤0.12)	S (≤0.12)
Benzylpenicillin	R (≥0.5)	R (≥0.5)	R (≥0.5)	R (≥0.5)	R (≥0.5)	R (≥0.5)
Oxacillin	R (≥4)	S (0.5)	S (0.5)	S (≤0.25)	R (≥4)	S (0.5)
Gentamicin	R (≥16)	S (≤0.5)	S (≤0.5)	S (≤0.5)	S (≤0.5)	S (≤0.5)
Ciprofloxacin	R (≥8)	S (≤0.5)	S (≤0.5)	S (≤0.5)	S (≤0.5)	S (≤0.5)
Levofloxacin	R (≥8)	S (0.25)	S (0.25)	S (≤0.12)	S (0.25)	S (0.25)
Moxifloxacin	R (≥8)	S (≤0.25)	S (≤0.25)	S (≤0.25)	S (≤0.25)	S (≤0.25)
Erythromycin	I (1)	R (≥8)	S (≤0.25)	R (≥8)	R (≥8)	S (≤0.25)
Clindamycin	S (≤0.25)	S (≤0.25)	S (≤0.25)	S (0.5)	S (≤0.25)	S (≤0.25)
Quinupristin/Dalfopristin	S (0.5)	S (≤0.25)	S (≤0.25)	S (0.5)	S (≤0.25)	S (≤0.25)
Linezolid	S (2)	S (2)	S (2)	S (2)	S (2)	S (2)
Vancomycin	S (1)	S (≤0.5)	S (≤0.5)	S (≤0.5)	S (≤0.5)	S (≤0.5)
Nitrofurantoin	S (≤16)	S (≤16)	S (≤16)	S (≤16)	S (≤16)	S (≤16)
Rifampicin	R (≥32)	S (≤0.5)	S (≤0.5)	S (≤0.5)	S (≤0.5)	S (≤0.5)
Trimethoprim	S (≤10)	S (≤10)	S (≤10)	S (≤10)	S (≤10)	S (≤10)

a R, resistant; S, susceptible; I, intermediate.

### Hinokitiol selectively enhances the antibacterial activity of tetracycline against S. aureus.

**FIG 1:**
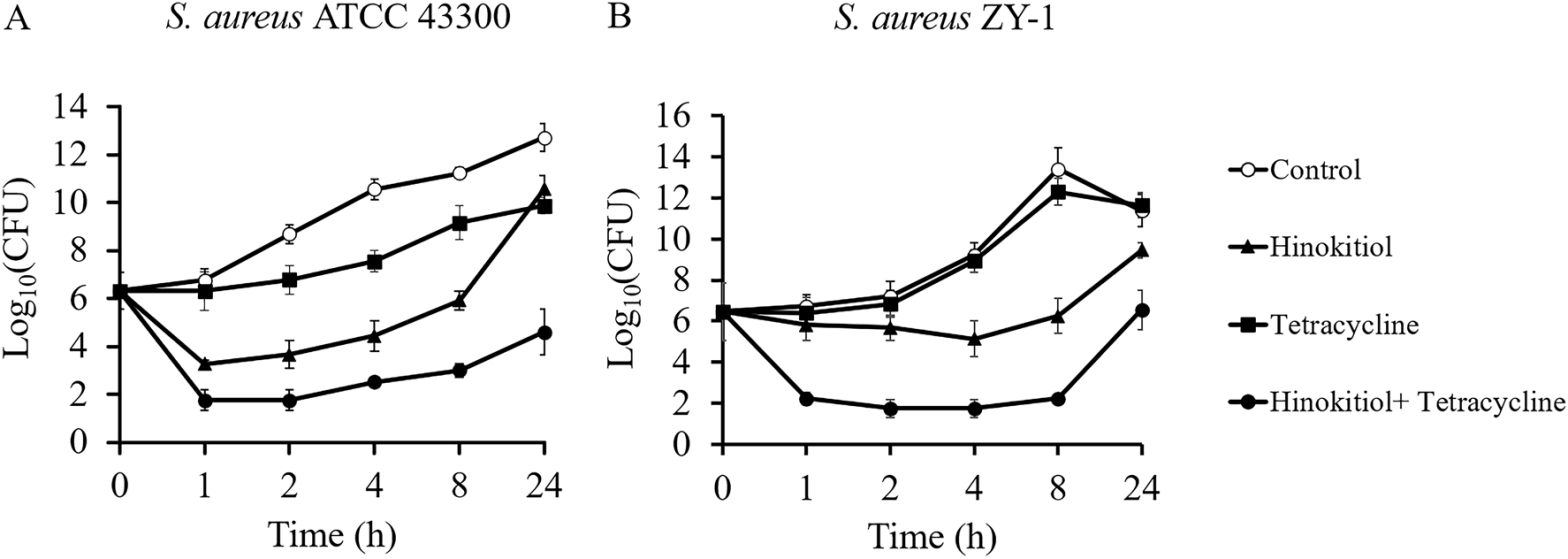
(A and B) Time-kill curves for S. aureus ATCC 43300 (A) and S. aureus ZY-1 (B). Data were obtained from three independent experiments presented as the average ± standard deviation (SD). Open circles, control (0.5% DMSO); closed triangles, 1/32 MIC of hinokitiol; closed squares, 1/4 MIC of tetracycline; closed circles, 1/32 MIC of hinokitiol combined with 1/4 MIC of tetracycline.

The MICs of hinokitiol in combination with tetracycline against various strains were assayed by the checkerboard method. As presented in Table 5, the combinations of hinokitiol with tetracycline were found to have no interaction against B. subtilis 168 and E. coli ATCC 35218 with fractional inhibitory concentration index (FICI) values of 1.25 and 1.25, respectively. Interestingly, the synergistic action of hinokitiol with tetracycline was observed against all tested S. aureus strains, regardless of whether they were tetracycline-resistant or tetracycline-sensitive ones. In addition, the combination of hinokitiol with tetracycline inhibited the paradoxical growth phenomenon. To further demonstrate the potentiating effect of hinokitiol on tetracycline activity, time-kill kinetics against two S. aureus strains (ATCC 43300 and MRSA ZY-1) at sub-MIC levels of hinokitiol and tetracycline were performed. As shown in Fig. 1, tetracycline in combination with hinokitiol displayed great antibacterial activity compared with monotherapy, which was consistent with the behavior revealed by the FICI analysis. A recent high-throughput screening of pairwise drug interactions revealed that more than 70% of the drug-drug interactions tested were species specific (19). Therefore, it is not surprising that the synergistic effect of hinokitiol with tetracycline against S. aureus rather than B. subtilis and E. coli was observed.

**TABLE 5:** MICs of hinokitiol in combination with tetracycline

Bacterial strain	MIC (μg/mL)			FICI	Category^*a*^
	Hinokitiol	Tetracycline	Combination (hinokitiol/tetracycline)		
B. subtilis 168	32	4	8/4	1.25	No interaction
E. coli ATCC 35218	16	0.5	4/0.5	1.25	No interaction
S. aureus ATCC 43300	32	0.25	1/0.03	0.156	Synergistic
S. aureus ATCC 29213	32	0.25	1/≤0.015	≤0.094	Synergistic
S. aureus ATCC 25923	8	0.25	2/0.03	0.375	Synergistic
S. aureus ZY-1 (MRSA)	32	16	1/0.25	0.047	Synergistic
S. aureus ZY-2	32	32	1/4	0.156	Synergistic
S. aureus ZY-3	32	32	1/8	0.281	Synergistic
S. aureus ZY-4	32	32	1/4	0.156	Synergistic
S. aureus ZY-5	32	0.25	1/0.015	0.094	Synergistic
S. aureus ZY-6	32	32	1/8	0.281	Synergistic

a FICI categories: ≤ 0.5, synergistic; > 0.5 to ≤ 1, additive; > 1 to < 4, no interaction; ≥ 4, antagonism.

### Hinokitiol enhances the efficacy of other tetracycline antibiotics against S. aureus.

Tetracyclines are broad-spectrum antibiotics with the characteristic naphthacene core comprising four aromatic rings. As one of the oldest classes of antimicrobials, tetracyclines represent a large and diverse group of active compounds (20), ranging from the naturally produced chlortetracycline to extended- and broad-spectrum semisynthetic derivatives of tetracycline, such as minocycline, tigecycline, and more recently, omadacycline. Tetracyclines exert their antibiotic activity by binding to the 30S ribosomal subunit, thus interfering with bacterial protein synthesis. To assess the ability of hinokitiol to increase susceptibility to other tetracycline antibiotics, its effect on tigecycline, minocycline, and chlortetracycline was tested using checkerboard assays. As shown in Table 6, the interaction of hinokitiol with tigecycline, minocycline, and chlortetracycline against ATCC 43300 was synergistic, with FICI values of 0.271, 0.151, and 0.0912, respectively. On MRSA ZY-1, a similar synergistic potency was observed. In general, the activity of all tested tetracycline antibiotics was enhanced in the presence of hinokitiol, with the respective MIC values being 4 to 64-fold lower than those in the absence of hinokitiol. In contrast, the combination of hinokitiol with ciprofloxacin or vancomycin exerted no synergistic activity against both strains of S. aureus.

**TABLE 6:** The MICs of hinokitiol with other tetracyclines alone and in combination

Strains and Antibiotics	MIC (μg/mL)		FICI	Category
	Alone	Combination		
S. aureus ZY-1 (MRSA)				
Hinokitiol	32	1	0.281	Synergistic
Tigecycline	0.125	0.03		
Hinokitiol	32	1	0.0462	Synergistic
Minocycline	2	0.03		
Hinokitiol	32	1	0.0625	Synergistic
Chlortetracycline	4	0.125		
Hinokitiol	32	4	0.625	Additive
Ciprofloxacin	32	16		
Hinokitiol	32	4	0.625	Additive
Vancomycin	1	0.5		
S. aureus ATCC 43300				
Hinokitiol	32	1	0.271	Synergistic
Tigecycline	0.125	0.03		
Hinokitiol	32	1	0.151	Synergistic
Minocycline	0.125	0.015		
Hinokitiol	32	1	0.0912	Synergistic
Chlortetracycline	0.25	0.015		
Hinokitiol	32	2	1.06	No interaction
Ciprofloxacin	0.5	0.5		
Hinokitiol	32	2	0.563	Additive
Vancomycin	1	0.5		

### Hinokitiol increases the intracellular accumulation of tetracycline.

**FIG 2:**
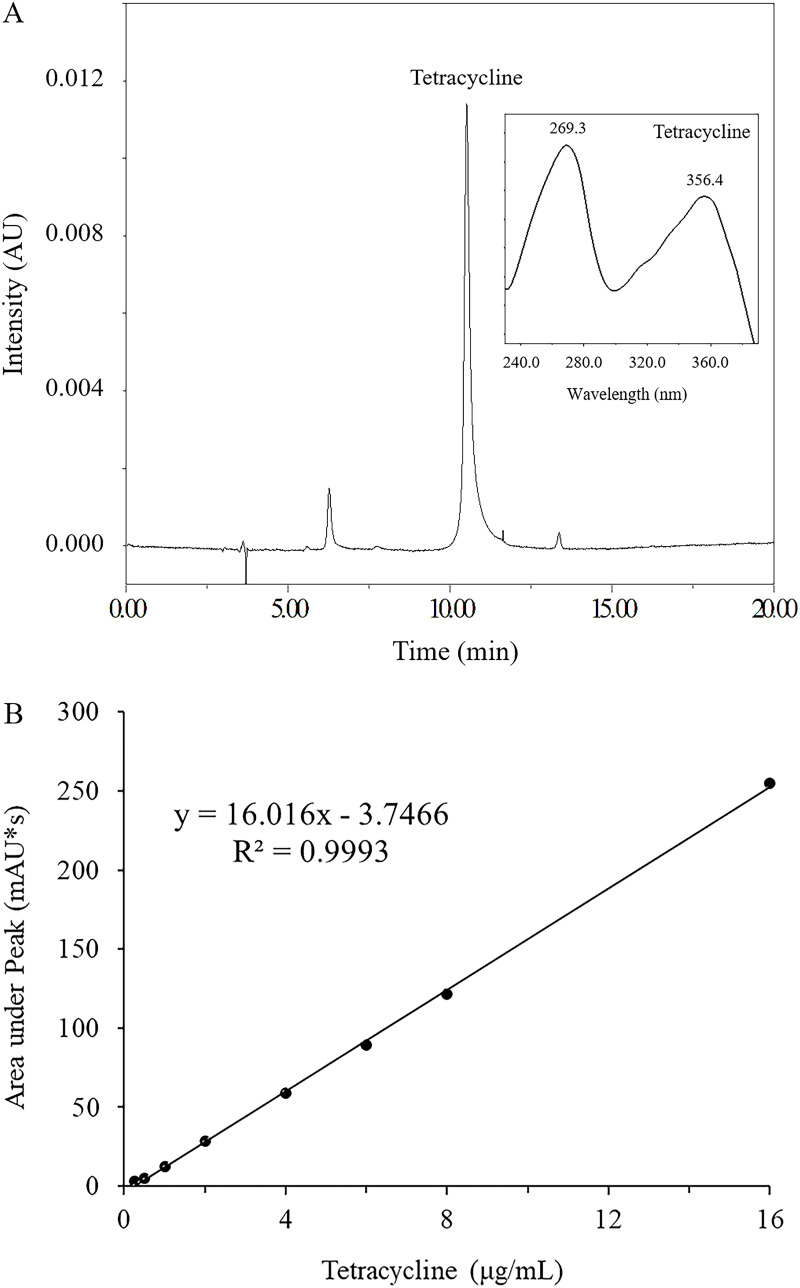
Evaluation of tetracycline by HPLC. (A) Typical HPLC chromatogram of tetracycline standard on the Agela Technologies C_18_ column (4.6 × 250 mm, 5 μm). (B) Standard samples containing increasing amounts of tetracycline using the same column as descried above. Concentration of standards was in the range of 0.25 to 16 μg/mL.

**FIG 3:**
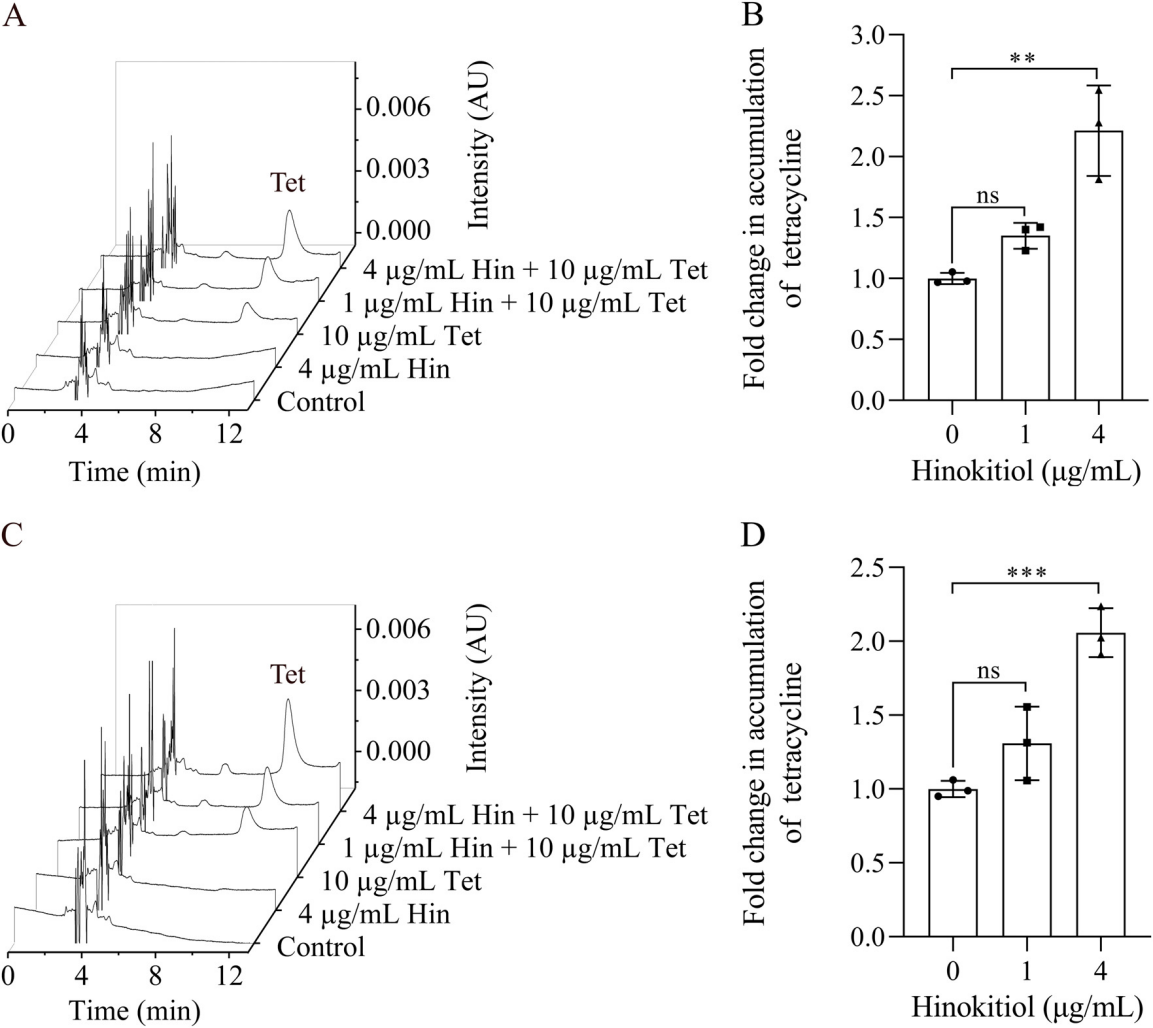
HPLC-based detection of intracellular tetracycline in S. aureus cells. (A to D) S. aureus ATCC 43300 (A and B) or MRSA ZY-1 (C and D) was treated with 10 μg/mL of tetracycline for 15 min with or without the presence of hinokitiol. Error bars indicate the standard deviation calculated from three biological replicates. Statistical significance was calculated with respect to S. aureus treated with 10 μg/mL of tetracycline. ***, *P* < 0.0005; **, *P* < 0.005; ns, no significance; control, treated with 0.5% DMSO; Hin, hinokitiol; Tet, tetracycline.

The high susceptibility of S. aureus to tetracycline in the presence of hinokitiol may be due to increased intracellular concentrations. To verify this possibility, the level of tetracycline in the cells of S. aureus was determined using high-performance liquid chromatography (HPLC). As shown in Fig. 2, standard tetracycline was eluted at 10.78 min on a C_18_ column, and a good linear correlation curve was obtained over the range of 0.25 to 16 μg/mL with a correlation coefficient of 0.999. When ATCC 43300 or MRSA ZY-1 was treated with tetracycline in the presence of 1 μg/mL hinokitiol, the intracellular concentration of tetracycline was increased about 30% in both strains compared to the cultures treated only with tetracycline (Fig. 3). A much higher intracellular concentration of tetracycline was observed when the concentration of hinokitiol was raised to 4 μg/mL, indicating that the accumulation of tetracycline was promoted by hinokitiol.

### Hinokitiol inhibits the efflux of EB without membrane depolarization.

**FIG 4:**
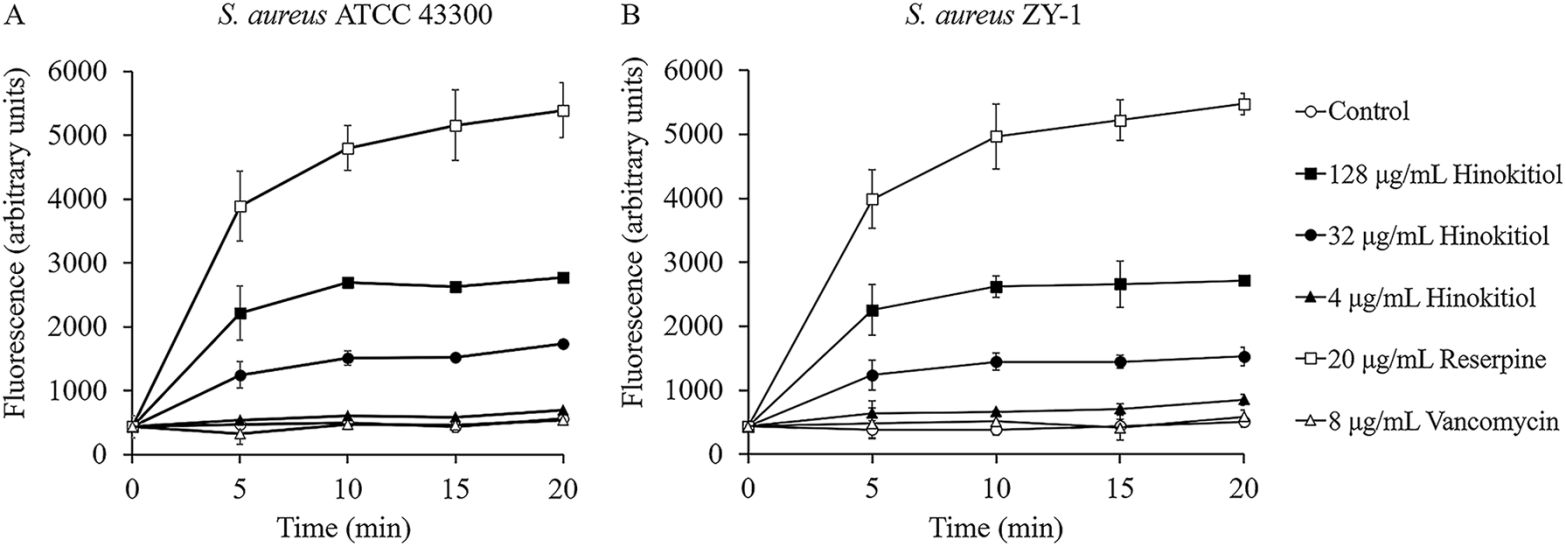
(A and B) Hinokitiol inhibits the efflux of ethidium bromide for S. aureus ATCC 43300 (A) and S. aureus ZY-1 (B). Data are presented as the mean ± SD of three independent experiments. Open circles, control (3.2% DMSO); closed squares, 128 μg/mL of hinokitiol; closed circles, 32 μg/mL of hinokitiol; closed triangles, 4 μg/mL of hinokitiol; open squares, 20 μg/mL of reserpine; open triangles, 8 μg/mL of vancomycin.

To determine whether an increased concentration of intracellular tetracycline in the presence of hinokitiol could be due to efflux pump inhibition, ATCC 43300 and MRSA ZY-1 EB accumulation assays were performed. EB is a substrate for many efflux pumps and is widely used to monitor the inhibition of drug efflux in given bacteria (21, 22). The fluorescence signal of EB is higher after its intercalation into bacterial DNA; thus, an increase in the fluorescence signal is an indication of its intracellular accumulation due to efflux inhibition. As an efflux pump inhibitor, reserpine allowed a significantly higher fluorescence increase of EB relative to the dimethyl sulfoxide (DMSO) control (Fig. 4). The addition of hinokitiol at 4 μg/mL (1/8× MIC) induced a weak accumulation of EB. However, at 32 or 128 μg/mL, hinokitiol significantly inhibited the EB efflux from both strains of S. aureus. As expected, the addition of vancomycin (8 μg/mL) had no effect on the accumulation of EB. These results were in agreement with the tetracycline accumulation assay results described above, suggesting that inhibiting efflux pump activity could be the mechanism by which hinokitiol increases the intracellular accumulation of tetracycline in S. aureus cells.

**FIG 5:**
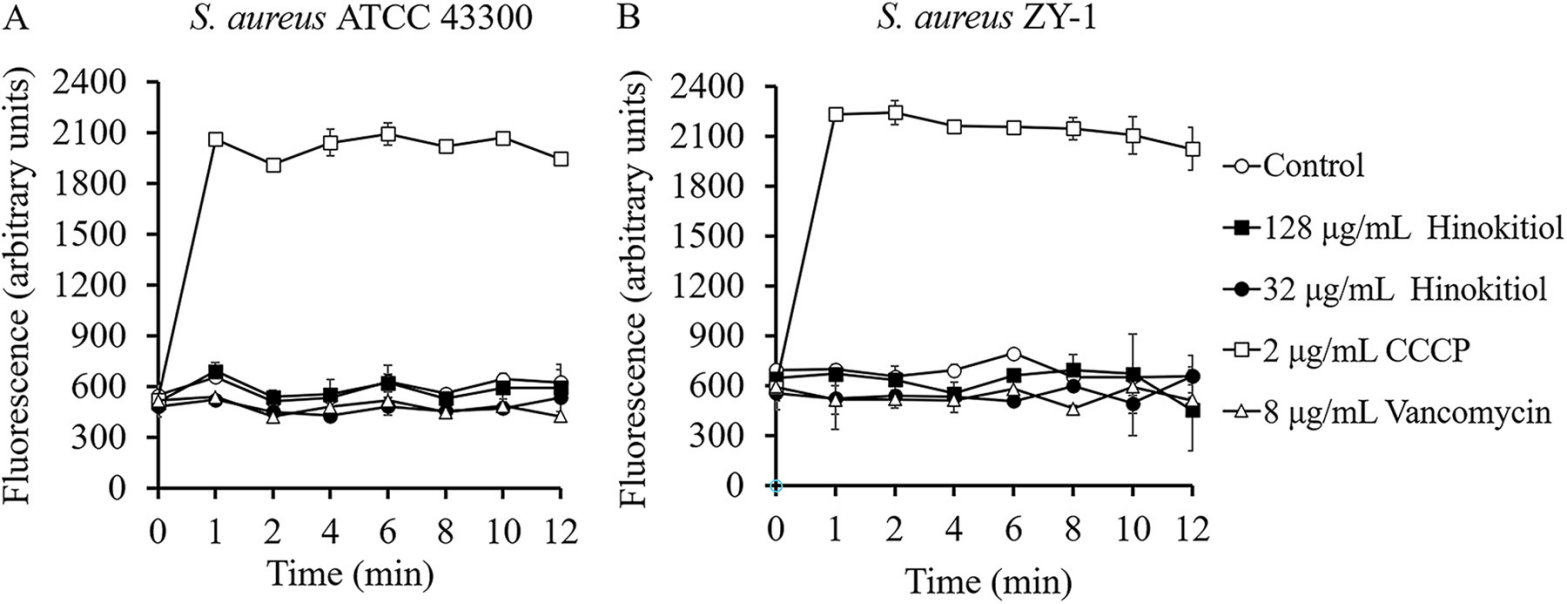
(A and B) Effect of hinokitiol on membrane potential of S. aureus ATCC 43300 (A) and S. aureus ZY-1 (B). Data from three independent experiments are presented as the mean ± SD. Open circles, control (3.2% DMSO); closed squares, 128 μg/mL of hinokitiol; closed circles, 32 μg/mL of hinokitiol; open squares, 2 μg/mL of CCCP; open triangles, 8 μg/mL of vancomycin.

Bacterial efflux pumps are typically energized by the proton motive force, which is reflected by the membrane potential (23, 24). Membrane potential assays using the fluorescent dye DiSC_3_(5) were performed to see if hinokitiol acts as an inhibitor of EB efflux by abolishing proton motive force. As shown in Fig. 5, the addition of hinokitiol up to 128 μg/mL to the suspensions of ATCC 43300 or MRSA ZY-1 caused no observable change in fluorescence compared to DMSO treatment. As expected, carbonyl cyanide m-chlorophenylhydrazone (CCCP) rapidly dissipated the membrane potential and led to a drastic increase in fluorescence in both strains. Hinokitiol had no effect on the membrane potential of S. aureus at the concentrations tested, implying that this agent inhibited drug efflux by means other than disrupting membrane potential.

## DISCUSSION

Antibiotic resistance has become a major threat to global health (1, 25). Plant-derived antimicrobial compounds with different mechanisms of action have received much attention due to their effectiveness against drug-resistant pathogens (2, 3, 26). Thousands of secondary plant compounds have been discovered and characterized as antimicrobial agents. However, most phytochemicals exhibit much higher MICs than antibiotics commonly used in clinical practice and cannot be used as monotherapy (2, 26). As a natural plant antibacterial agent widely used in oral care and therapeutic products (12, 15, 27), hinokitiol suffers from the same problem, especially against some bacteria, such as S. aureus. At present, there is very little information about the synergistic antibacterial activity between hinokitiol and other compounds, except that the combination of hinokitiol and zinc oxide enhanced killing activity against clinically isolated staphylococci (12). In this study, we investigated the synergistic potential of hinokitiol in combination with conventional antibiotics and found that hinokitiol selectively enhanced the antibacterial activity of tetracycline antibiotics against S. aureus.

S. aureus is a common opportunistic pathogen that causes a variety of human diseases, ranging from mild skin and soft tissue infections to life-threatening illnesses such as bacteremia, endocarditis, and septic shock (28–30). Infections caused by this bacterium remain a challenging public health problem due to the emergence and wide spread of drug-resistant strains. As one of the “big four” classes of antibiotics, tetracyclines have been used for the treatment of infections caused by a variety of pathogens, including Gram-positive and Gram-negative bacteria (20, 31, 32). However, the increased resistance to these antibiotics together with their toxicity have limited the use of these groups of antibiotics in the clinic (20, 31, 32). In fact, tetracyclines are currently used as antistaphylococcal agents mainly in the treatment of skin or skin structure infections (33, 34). Hinokitiol effectively potentiated the antibacterial activity of tetracyclines against drug-susceptible or -resistant S. aureus, suggesting that this phytochemical is a promising compound for the development of new synergists with the potential to extend the pharmacological action of tetracyclines.

It has been reported that two drugs will be synergistic if one antibiotic helps another drug’s availability in bacterial cells (19, 35). HPLC-based detection of tetracycline demonstrated that hinokitiol caused a dose-dependent increase in the accumulation of tetracycline, indicating that this phytochemical helped tetracycline get inside S. aureus cells. Efflux pumps play an important role in the accumulation of tetracycline in bacterial cells (36). Many compounds that act as pump inhibitors have been reported to increase drug accumulation in bacterial cells by decreasing the drug’s efflux (37–39). Our results indicated that hinokitiol promoted the accumulation of EB in both strains of S. aureus without membrane depolarization, implying that the phytochemical may act as an efflux pump inhibitor.

An intriguing phenomenon was that the potentiation of hinokitiol was observed only on tetracyclines against S. aureus but not on other antibiotics (ciprofloxacin and vancomycin) or against other bacteria (E. coli and B. subtilis). This observation could possibly be explained by the inhibition of hinokitiol against the intrinsic tetracycline-specific efflux pumps of S. aureus. Antibiotic efflux pumps widely distributed in various bacterial species are membrane transport proteins that can be specific for a single substrate (for example, TetA in E. coli selectively excludes tetracycline) or capable of pumping out a wide range of structurally diverse substrates (for example, NorA can extrude several distinct classes of antibiotics) (40). The latest tally shows that a total of 36 distinct tetracycline-specific efflux pumps have been characterized in Gram-negative and Gram-positive bacteria (41). The most common tetracycline-specific efflux pumps are members of the major facilitator superfamily (MFS) of transporters (20, 36). In staphylococci, three MFS tetracycline-specific efflux pumps with 14 transmembrane segments called TetL, TetK, and Tet38 have been described (20, 42, 43). The gene of TetK or TetL is generally found on plasmids, while Tet38 is a chromosomally encoded intrinsic efflux pump that is present in susceptible as well as resistant S. aureus (31). In this study, the synergistic effect of the hinokitiol-tetracycline combination against resistant and susceptible S. aureus with no significant difference between their FICIs might be due to its inhibition of an intrinsic efflux pump such as Tet38 or other unknown transporters. However, whether the selectively enhanced antibacterial effects of hinokitiol on tetracycline are related to its specific efflux pump inhibition activity remains to be further clarified.

In conclusion, as a low-toxicity natural product, hinokitiol has shown synergistic activity in combination with tetracycline antibiotics against S. aureus, including tetracycline-resistant strains and MRSA. The accumulation of tetracycline promoted by hinokitiol may partially explain this phenomenon. Although further experiments are necessary to explore the clinical potential of hinokitiol in more detail, the hinokitiol-tetracycline combination offers a promising alternative approach for the treatment of skin infections caused by refractory S. aureus.

## MATERIALS AND METHODS

### Chemicals and bacterial strains.

Hinokitiol (>99.0% GC content), formate (≥98% HPLC), and chlortetracycline were purchased from Aladdin Bio-Chem Technology Co., Ltd. (Shanghai, China). Vancomycin, ciprofloxacin, erythromycin, and minocycline were purchased from Shanghai Yuanye Bio-Technology Co., Ltd. (China). Tetracycline and tigecycline were purchased from Shanghai Macklin Biochemical Co., Ltd. (China). Acetonitrile (HPLC) was purchased from Tedia Co., Inc. (USA).

S. aureus ATCC 43300, S. aureus ATCC 29213, S. aureus ATCC 25923, E. coli ATCC 35218, and B. subtilis 168 were preserved in our laboratory. Clinical isolates, including S. aureus ZY-1, S. aureus ZY-2, S. aureus ZY-3, S. aureus ZY-4, S. aureus ZY-5, and S. aureus ZY-6 were obtained from patients at the First Affiliated Hospital of Zhejiang University, Hangzhou, China. All bacterial strains were grown routinely on MH medium (Oxoid, UK).

### Determination of MIC.

The MIC was evaluated using a broth microdilution method (44). Overnight cultures of bacteria were seeded at ~5 × 10^5^ cells per well in a 96-well plate containing MH broth with various concentrations of each test agent. The MIC was defined as the lowest concentration that completely prevented visible growth after incubation at 37°C for 18 to 20 h. The MIC of the drugs alone and in combination were recorded, and the FICI was used to calculate and judge the combined effect of two drugs. The FICIs were calculated using the following equation: FICI = FIC_A_ + FIC_B_; FIC_A_ = MIC of drug A in combination/MIC of drug A alone; FIC_B_ = MIC of drug B in combination/MIC of drug B alone. The FICIs were obtained from three independent experiments and interpreted as previously described: synergy (FICI ≤ 0.5), additive (0.5 < FICI ≤ 1), no interaction (1< FICI ≤ 4), antagonism (FICI > 4) (45).

### Effect of pH and inoculum size.

The effect of changes in the inoculation size and the pH of the medium on the MIC of hinokitiol was studied with two strains of S. aureus (46). The inoculum size changed from 10^4^ to 10^6^ CFU/mL, while the initial pH of the medium was adjusted to 6.5, 7.0, and 7.5. All experiments were performed with three replicates.

### Time-kill assay.

The time-kill kinetics of S. aureus ATCC 43300 and S. aureus ZY-1 were determined as previously reported (44). Overnight culture of the strain was adjusted to ~10^6^ CFU/mL with fresh MH medium. Different agents with subinhibitory concentrations of hinokitiol and tetracycline were added to the liquid culture medium. The same volume of DMSO was added as a control. All tubes were incubated at 37°C with shaking for 24 h. Aliquots (100 μL) were taken from each tube at 0, 1, 2, 4, 8, and 24 h, serially diluted (1:10) with cold saline, and spotted on LB agar plates. Viable bacteria were quantitated after 24 h of incubation at 37°C. The experiment was performed in triplicate.

### Tetracycline accumulation assay.

Overnight cultures of S. aureus were harvested by centrifugation (7,000 rpm, 5 min) at 4°C. The pellets were resuspended in 100 mL LB broth to an optical density at 600 nm (OD_600_) of 0.5 and treated with 10 μg/mL of tetracycline for 15 min with or without the presence of hinokitiol. The cells were harvested by centrifugation and then washed once in ice-cold water. Cell pellets were resuspended in 3 mL methanol and scraped up and down until the cell pellets were completely disrupted (47). After being centrifuged at 12,000 rpm for 10 min, the supernatant was transferred into centrifugal tubes and concentrated up to dryness using a vacuum centrifugal concentrator RC10-10 (Thermo Electron Co., USA) at 50°C. The residue was dissolved in 200 μL of DMSO, and subsequently analyzed by reversed-phase HPLC. Final tetracycline accumulation was calculated by normalization with the dry cell weight.

A Waters HPLC e2695 separation module with a 2998 photodiode array (PDA) detector was employed (Waters Co., USA) to analyze the samples. The Agela Technologies C_18_ column (4.6 × 250 mm, 5 μm) was employed for chromatographic separation. The mobile phase consisting of solvent A (acetonitrile) and solvent B (0.1% formic acid) had a flow-rate of 1.00 mL/min. The gradient elution program was as follows: 0 min, A 10% and B 90%; 5 min, A 10% and B 90%; 20 min, A 30% and B 70%; 23 min, A 30% and B 70%; 24 min, A 10% and B 90%. The detection wavelength was 355 nm, and the column temperature was 35°C (48).

### EB fluorescence assay.

The efflux pump inhibition test was assessed using EB as described earlier with suitable modifications (49). S. aureus was cultured to the mid-log phase and harvested by centrifugation. The bacterial cells were washed once with 20 mM potassium phosphate buffer (pH 7.2) and resuspended to an OD_600_ of 0.2 in the buffer containing 10 mM glucose. After preincubatiopn with different agents for 15 min at 37°C, 1 μg/mL EB was added to the cell suspensions, and the fluorescence was measured at excitation and emission wavelengths of 480 nm and 610 nm, respectively.

### Determination of membrane potential.

The effect of hinokitiol on bacterial membrane potential was determined using the membrane potential-sensitive cyanine dye DiSC_3_(5) as previously described (50). Bacteria grown to the logarithmic growth phase were harvested, washed three times, and resuspended in a buffer containing 10 mM potassium phosphate, 5 mM MgSO_4_, and 250 mM sucrose (pH 7.0). After adjusting the OD_600_ to 0.2, 1 μM DiSC_3_(5) was added to the bacterial suspension. The cells were then added to 96-well plates and allowed to stabilize before the test agents were added. The excitation wavelength was 620 nm, and the emission wavelength was 685 nm.
